# Multifactorial resistance to aminopeptidase inhibitor prodrug CHR2863 in myeloid leukemia cells: down-regulation of carboxylesterase 1, drug sequestration in lipid droplets and pro-survival activation ERK/Akt/mTOR

**DOI:** 10.18632/oncotarget.6169

**Published:** 2015-10-19

**Authors:** Sue Ellen Verbrugge, Marjon Al, Yehuda G. Assaraf, Sarah Kammerer, Durga M.S.H. Chandrupatla, Richard Honeywell, Rene P.J. Musters, Elisa Giovannetti, Tom O'Toole, George L. Scheffer, David Krige, Tanja D. de Gruijl, Hans W.M. Niessen, Willem F. Lems, Pieternella A. Kramer, Rik J. Scheper, Jacqueline Cloos, Gert J. Ossenkoppele, Godefridus J. Peters, Gerrit Jansen

**Affiliations:** ^1^ Department of Rheumatology, Amsterdam Rheumatology and Immunology Center, VU University Medical Center, Amsterdam, The Netherlands; ^2^ The Fred Wyszkowsky Cancer Research Laboratory, Faculty of Biology, The Technion-Israel Institute of Technology, Haifa, Israel; ^3^ Department of Medical Oncology, VU University Medical Center, Amsterdam, The Netherlands; ^4^ Department of Physiology, VU University, Amsterdam, The Netherlands; ^5^ Department of Molecular Cell Biology, VU University, Amsterdam, The Netherlands; ^6^ Departments of Pathology and Cardiac Surgery, ICaR-VU, VU University Medical Center, Amsterdam, The Netherlands; ^7^ Chroma Therapeutics Ltd, Abingdon, United Kingdom; ^8^ Isala Hospital, Zwolle, The Netherlands; ^9^ Department of Pediatric Oncology/Hematology, VU University Medical Center, Amsterdam, The Netherlands; ^10^ Department of Hematology, VU University Medical Center, Amsterdam, The Netherlands; ^11^ Present address: Department of Clinical Chemistry, UMCU, Utrecht, The Netherlands; ^12^ Present address: Institute of Biophysics, Medical University of Graz, Graz, Austria; ^13^ Present address: Immunocore Ltd, Oxford, UK

**Keywords:** aminopeptidase, carboxylesterase, lipid droplets, mTOR, rapamycin

## Abstract

Aminopeptidase inhibitors are receiving attention as combination chemotherapeutic agents for the treatment of refractory acute myeloid leukemia. However, the factors determining therapeutic efficacy remain elusive. Here we identified the molecular basis of acquired resistance to CHR2863, an orally available hydrophobic aminopeptidase inhibitor prodrug with an esterase-sensitive motif, in myeloid leukemia cells. CHR2863 enters cells by diffusion and is retained therein upon esterase activity-mediated conversion to its hydrophilic active metabolite drug CHR6768, thereby exerting amino acid depletion. Carboxylesterases (CES) serve as candidate prodrug activating enzymes given CES1 expression in acute myeloid leukemia specimens. We established two novel myeloid leukemia sublines U937/CHR2863(200) and U937/CHR2863(5uM), with low (14-fold) and high level (270-fold) CHR2863 resistance. The latter drug resistant cells displayed: (i) complete loss of CES1-mediated drug activation associated with down-regulation of CES1 mRNA and protein, (ii) marked retention/sequestration of the prodrug, (iii) a substantial increase in intracellular lipid droplets, and (iv) a dominant activation of the pro-survival Akt/mTOR pathway. Remarkably, the latter feature coincided with a gain of sensitivity to the mTOR inhibitor rapamycin. These finding delineate the molecular basis of CHR2863 resistance and offer a novel modality to overcome this drug resistance in myeloid leukemia cells.

## INTRODUCTION

Aminopeptidases (AP) play an essential role in protein and peptide homeostasis by regulating their modification, maturation, activation and degradation [1]. Small peptides may either be completely hydrolyzed to amino acids for renewed protein biosynthesis or trimmed for major histocompatibility class I presentation to initiate CD8^+^ T cell-mediated immune responses [2]. Moreover, plasma membrane-associated APs such as aminopeptidase N (CD13) can serve as cell function mediators, e.g. in signal transduction pathways in immune cells [3, 4] or endothelial cells [5]. Consequently, the relevance of APs extends to malignant and (chronic) inflammatory diseases [6-9] and may thus provide opportunities for therapeutic interventions [10]. In this context, bestatin represented the first prototypic AP-inhibitor tested in the clinical setting and displayed immune-modulatory properties through suppression of the production of pro-inflammatory cytokines by activated macrophages [11] as well as anti-proliferative activity in lung cancer [12] and acute myeloid leukemia [13]. Building on bestatin as a direct AP inhibitor, prodrug versions of AP-inhibitors are currently being evaluated, of which Tosedostat (CHR2797) [14] displayed promising clinical activity against acute myeloid leukemia [15-17], multiple myeloma [18] and solid tumors [19, 20]. As a hydrophobic prodrug Tosedostat harbors a cyclopentyl ester that requires intracellular cleavage by (carboxyl) esterase activity [21] to yield a hydrophilic acid form that enhances its intracellular retention. Since carboxylesterases are highly expressed in myelomonocytic leukemia cells [22], this may underlie Tosedostat's activity towards AML. Tosedostat blocks multiple APs, including aminopeptidase N (CD13), leucine aminopeptidase and puromycin-specific aminopeptidase [14]. These inhibitory activities were mediated by its conversion to its active metabolite, while the latter displayed a potent inhibitory activity against leukotriene A4 hydrolase [14]. Upon AP inhibition, Tosedostat provoked an intracellular amino acid depletion and suppressed cell growth as part of an amino acid deprivation response as well as inhibition of mTOR [14, 23].

As AP prodrugs with esterase motifs are being evaluated in the clinic, we herein addressed the important question as to whether prolonged exposure to these classes of drug would provoke the onset of drug resistance, and if so, elucidate the molecular basis of this drug resistance. We report that human myelomonocytic U937 cells acquired resistance to CHR2863, an orally available close structural analogue of Tosedostat/CHR2797, via multifactorial mechanisms involving down-regulation of carboxylesterase-1 and its association with lipid droplets, prodrug sequestration and lack of conversion to its active metabolite, and activation of ERK/Akt/mTOR pro-survival pathways. We further show that the latter could be exploited to efficiently overcome CHR2863 drug resistance using mTOR-targeted drugs like rapamycin.

## RESULTS

### Development of CHR2863 resistance and cross-resistance profile

**Figure 1:**
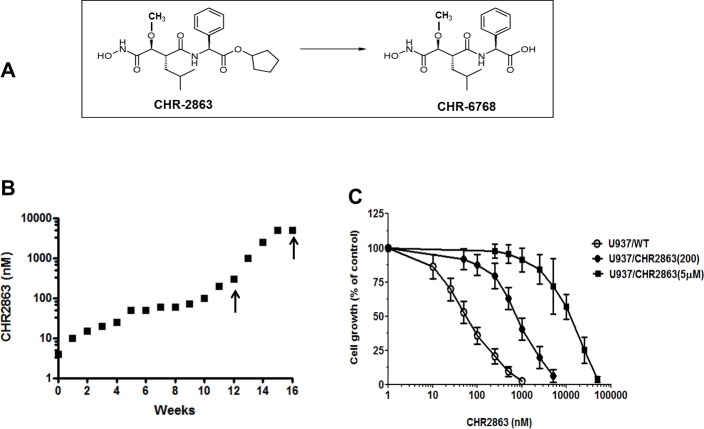
**A.** Chemical structure of the aminopeptidase inhibitor prodrug CHR2863 with an esterase motif and its acid metabolite CHR6768. **B.** Time line for acquisition of resistance to CHR2863 in U937 cells. Two isolates (indicated by arrows) were selected for further characterization; U937 cells grown in the presence of 200 nM CHR2863 (U937/CHR2863(200) and U937 cells grown in the presence of 5 μM CHR2863 (U937/CHR2863(5μM). **C.** Dose response curve of for growth inhibition by CHR2863 for U937/WT, U937/CHR2863(200), and U937/CHR2863(5μM) cells. Results depicted are the mean ± SD of 7-10 separate experiments

CHR2863, a close structural analogue of Tosedostat (CHR2797) with a methoxy-group in the hydroxycarbamoyl moiety (Figure 1A) was administered to human myelomonocytic U937 cells in gradually (stepwise) increasing concentrations over a period of 4-6 months. During this multiple step selection U937 cells acquired resistance to CHR2863 (Figure 1B) and two sublines were isolated for further characterization; one with a low level resistance grown at a concentration of 200 nM CHR2863 (U937/CHR2863(200)) and another with a high level resistance grown at a concentration of 5μM CHR2863 (U937/CHR2863(5μM)). Dose response curves for CHR2863-induced growth inhibition in parental U937/WT cells and the two sublines are shown in Figure 1C. Resistance factors were found to be 13.7-fold and as high as 270-fold for U937/CHR2863(200) and U937/CHR2863(5μM) cells, respectively (Table 1).

**Table 1:** Growth inhibitory effects of aminopeptidase inhibitor prodrug CHR2863 and other (pro) drugs for parental U937 myelomonocytic cells (U937/WT) and sublines of U937 cells with acquired resistance to CHR2863

	IC_50_^&^		
Drug	U937/WT	U937/CHR2863(200)	U937/CHR2863(5μM)
**CHR2863 (nM)**	52 ± 16	713 ± 212*** *(13.7*)	14,047 ± 5,521*** *(270*)
**Bestatin (μM)**	149 ± 21	151 ± 45 *(1.0)*	177 ± 12 *(1.2)*
**CHR5346 (μM)**	8.4 ± 1.4	9.3 ± 1.1 *(1.1)*	13.7 ± 4.2 *(1.6)*
**CHR2875 (nM)**	152 ± 30	84 ± 10*** *(0.55)*	158 ± 33 *(1.0)*
**CPT-11/Irinotecan (μM)**	1.37 ± 0.29	0.60 ± 0.18** *(0.44)*	0.45 ± 0.10** *(0.33)*
**Capecitabine (Xeloda) (mM)**	1.5 ± 0.3	3.3 ± 0.3** *(2.2)*	3.4 ± 0.3** *(2.3)*
**Ara-C (nM)**	49 ± 14	59 ± 24 *(1.2)*	23 ± 10** *(0.47)*
**Daunorubicin (nM)**	16 ± 2	15 ± 1 *(1.0)*	15 ± 2 *(1.0)*
**Bortezomib (nM)**	3.5 ± 0.5	5.8 ± 1.8* *(1.7)*	4.1 ± 0.8 *(1.2)*
**Carfilzomib (nM)**	4.9 ± 1.3	7.3 ±1.2* *(1.5)*	6.9 ± 1.3 *(1.4)*

& Cell growth inhibition was determined after 72 hrs drug exposure and results depicted are the mean IC_50_ values of 4-7 independent experiments ± S.D. IC_50_ is defined as drug concentration resulting in 50% growth inhibition compared to control. Values between brackets represent Resistance Factor, defined as the ratio of IC_50_ value of U937/CHR2863-resistant cells over IC_50_ of parental U937/WT cells. Statistics

* p<0.05

** p<0.01

*** p<0.001 (resistant cells *vs* wild type cells).

Cross-resistance profiling for other selected (pro)drugs (Table 1) showed lack of cross-resistance to the direct AP-inhibitor bestatin and CHR5346 (a non-cleavable analogue of CHR2797), suggesting that alterations in AP-levels do not contribute to CHR2863 resistance. CHR2863-resistant cells also retained sensitivity to CHR2875, an HDAC-inhibitor prodrug [21]. Interestingly, CHR2863-resistant cells displayed a collateral hypersensitivity of 2-3 fold to the topoisomerase inhibitor prodrug CPT-11/irinotecan, but were 2-fold less sensitive to the 5-fluorouracil prodrug Capecitabine/Xeloda. CHR2863-resistant cells retained sensitivity to cytarabine and daunorubicin, two drugs which are usually combined with Tosedostat/CHR2797 in AML therapy [15]. Finally, growth inhibitory effects of two proteasome inhibitors Bortezomib (Velcade) and carfilzomib [24], functioning upstream of APs in protein degradation pathways, were unaltered in CHR2863-resistant cells.

Examination of the stability of the drug resistance phenotype revealed that in the absence of the selecting drug, U937/CHR2863(200) cells rapidly lost (within 1 month) their CHR2863 resistance. In contrast, U937/CHR2863(5μM) cells retained their drug resistance phenotype in the absence of CHR2863 for > 3 months, thereby establishing a genetically stable resistance phenotype (Supplementary Figure S1).

As an initial approach to unravel the molecular basis underlying CHR2863 resistance, we explored whether drug extrusion via multidrug resistance (MDR)-related drug efflux transporters [25] could be involved as they can extrude a broad spectrum of hydrophobic drugs (e.g. CHR2863) or hydrophilic drugs (e.g. CHR6768, the acid form of CHR2863). Western blot analysis of a series of drug efflux transporters revealed either no detectable expression of these MDR efflux transporters (P-glycoprotein, MRP2 and MRP3) or no differential expression (MRP1, MRP5 and BCRP) in U937/WT and a series of CHR2863-resistant U937 cells (Supplementary Figure S2). Of note, expression of MRP4 was gradually increased in U937 cells with increasing levels of CHR2863 resistance. Elevated levels of MRP4 were, however, not directly accountable for CHR2863 resistance as co-incubation with an established inhibitor of MRP4 (i.e. MK571) had no reversal effect on CHR2863 resistance (results not shown). Together, these results and cross-resistance profiling point to a non-classical mechanism of CHR2863 resistance.

### Intracellular sequestration CHR2863 and lack of its conversion to the active metabolite in U937/CHR2863(5μM) cells

**Figure 2:**
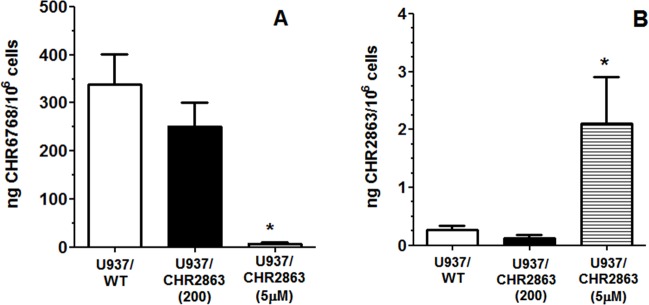
**A.**Conversion of CHR2863 to CHR6768 and **B.** retention of CHR2863 in U937/WT, U937/CHR2863(200), and U937/CHR2863(5μM) cells after 6 hr exposure to 6 μM CHR2863. Results are expressed as ng/10^6^ cells and represent the mean ± SE of 7-9 separate experiments. (*): *p* < 0.001

Since conversion of CHR2863 to the hydrophilic acid metabolite CHR6768 is essential for its pharmacological activity, we determined this capacity in U937/WT and U937/CHR2863 cells. U937/WT displayed a proficient and linear (not shown) conversion of CHR2863 into CH6768 (338 ± 63 ng/10^6^ cells) over a 6 hr exposure to 6 μM CHR2863 (Figure 2A). Under these conditions, U937/CHR2863(200) cells displayed a 24% reduced conversion to CHR6768 (251 ± 47 ng drug/10^6^ cells) as compared to U937/WT cells. Strikingly, however, conversion of CHR2863 to CHR6768 in U937/CHR2863(5μM) cells was essentially completely abolished (7.3 ± 2.2 ng drug/10^6^ cells, thereby losing 98% of parental U937/WT enzymatic conversion capacity. Additionally, beyond the conversion to the active metabolites, we also determined the levels of the CHR2863 prodrug retained in these three myeloid leukemia cell lines (Figure 2B). In U937/WT and U937/CHR2863(200) cells, absolute intracellular levels of CHR2863 were 3 orders of magnitude lower than those of CHR6768, being 0.27 ± 0.07 ng CHR2863 /10^6^ cells and 0.12 ± 0.05 ng CHR2863/10^6^ cells), respectively. Remarkably, U937/CHR2863(5μM) cells retained significantly higher levels (8-17 fold) of prodrug (2.0 ± 0.8 ng CHR2863/10^6^ cells) compared to U937/WT and U937/CHR2863(200) cells, thus suggesting sequestration of the prodrug in these cells and evasion from conversion to CHR6768.

As a comparison we determined the cellular levels of the HDAC prodrug inhibitor CHR2875 and its active metabolite CHR2880 after 6 hours of exposure to 6 μM CHR2875. Of note, absolute levels of CHR2880 were approximately 100-fold lower than for CHR6768, but no significant differences in levels of CHR2875 and CHR2880, respectively, were observed for U937/WT (0.43 ± 0.18 and 3.6 ± 0.2 ng/10^6^ cells), U937/CHR2863(200) cells (0.55 ± 0.15 and 3.0 ± 1.6 ng/10^6^ cells) and U937/CHR2863(5μM) cells (0.66 ± 0.05 and 4.3 ± 2.5 ng/10^6^ cells), being consistent with a comparable drug sensitivity profile between the parent and drug resistant sublines shown in Table 1.

These results indicate that at least for U937/CHR2863(5μM) cells, CHR2863 resistance is associated with intracellular drug sequestration along with a markedly impaired conversion to its acid metabolite CHR6768.

### Carboxylesterase expression in myeloid leukemia cells

**Figure 3:**
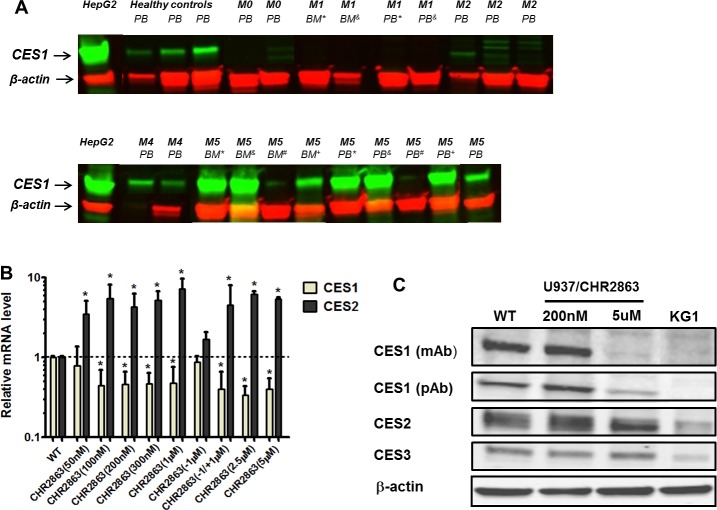
Carboxylesterase 1 and 2 expression in acute myeloid leukemia (AML) cells and CHR2863-resistant U937 cells **A.** CES1 expression (Western blot) in FAB sub-classified (M0, M1, M2, M4, M5) acute myeloid leukemia cells at diagnosis. Superscript symbols refer to matched peripheral blood (PB) and bone marrow (BM) samples. CES1 expression in peripheral blood of healthy controls is depicted for reference. Hep-G2 hepatoma cells serve as control for CES1 and CES2 protein expression. CES2 expression was not observed in the indicated AML samples (not shown). **B.** mRNA expression of CES1 and CES2 in multiple isolates of CHR2863-resistant U937 cells, including U937/WT, U937/CHR2863(200), and U937/CHR2863(5μM) cells. U937/CHR2863(w/o 1 μM) without CHR2863 for 2 weeks and U937/CHR2863 (w/o 1μM/+1μM) rechallenged with 1 μM CHR2863 for 2 weeks. Mean (± SD) of 3-4 experiments performed in triplicate). (*): *p* < 0.05. **C.** Western blots of CES1 (by two different antibodies), CES2 and CES3 protein expression in U937/WT, U937/CHR2863(200), and U937/CHR2863(5μM) cells. KG1 cells served as negative control for CES1 expression [21].

Since carboxylesterases have an established and prominent role as drug metabolizing enzymes [21, 26-28], we examined the expression levels of two of its family members; carboxylesterase 1 (CES1) and carboxylesterase 2 (CES2), in bone marrow and/or peripheral blood cells of AML patients at diagnosis. Consistent with gene expression profiling data of AML blasts cells [22] we observed differential expression of CES1 being more prominent in (myelo) monocytic M4 and M5 FAB classification subtypes than other subtypes (Figure 3A). Of note, CES1 expression was consistently high in matched peripheral blood and bone marrow cells of M5 patients. CES2 expression was not detectable by Western blot analysis of AML cells as compared to HepG2 hepatoma cells serving as a positive control (data not shown).

### Down-regulation of CES1 and upregulation of CES2 in U937/CHR2863 cells

We next examined whether CES1 and/or CES2 were involved in CHR2863 conversion and drug resistance. Analysis of CES1 and CES2 mRNA levels in U937/CHR2863 cells showed a remarkable down-regulation of CES1 (3-5 fold), being mirrored by an up-regulation of CES2 mRNA (Figure 3B). Interestingly, when U937/CHR2863(1μM) cells were grown in drug-free medium for 2 weeks, this normalized CES1 and CES2 mRNA to U937/WT levels, but a rechallenge with 1 μM CHR2863 reinforced down-regulation of CES1 and up-regulation of CES2 mRNA (Figure 3B). To understand whether these dynamics were resistance-induced or could also be noted after pulse exposure (0-6 hrs) to CHR2863, U937/WT cells were exposed to either 50 nM CHR2863 (IC_50_ concentration) or 6 μM CHR2863 (used for metabolite conversion experiments). Both conditions induced CES1 mRNA down-regulation (up to 3-fold) and a comparable up-regulation of CES2 mRNA (Supplementary Figure S3A/B). Since U937/CHR2863(200nM) and U937/CHR2863(5μM) cells were already down-regulated in CES1 and up-regulated in CES2 mRNA expression, pulse exposure to 6 μM CHR2863 only modestly increased this differential or had no additional effect, respectively (Supplementary Figure S3C/D).

We next examined whether differences in CES1 and CES2 mRNA were also reflected at the protein level. CES1 protein levels were not significantly altered in U937/CHR2863(200nM) compared to U937/WT cells, but U937/CHR2863(5μM) cells displayed a markedly down-regulated CES1 expression (Figure 3C). Rather, CES2 protein expression was modestly increased in both CHR2863-resistant cells compared to U937/WT cells, whereas expression of another CES homologue, CES3, was unaltered (Figure 3C).

CES1 knockdown by using CES1 siRNA was performed in U937/WT cells which resulted in 85% reduction of CES1 mRNA expression and a 35% reduced conversion of CHR2863 to its active metabolite in U937/WT controls. This reduction, however, had no significant impact on growth inhibitory effects of CHR2863 (not shown), suggesting that knock down of CES1 did not reach a critical level to fully impair CHR2863 conversion and thereby confer resistance. Together, these studies indicate that CHR2863 induces differential response in CES1 and CES2 expression which, upon prolonged exposure, may contribute to acquisition of CHR2863 resistance.

### CES1 association with lipid droplets

**Figure 4:**
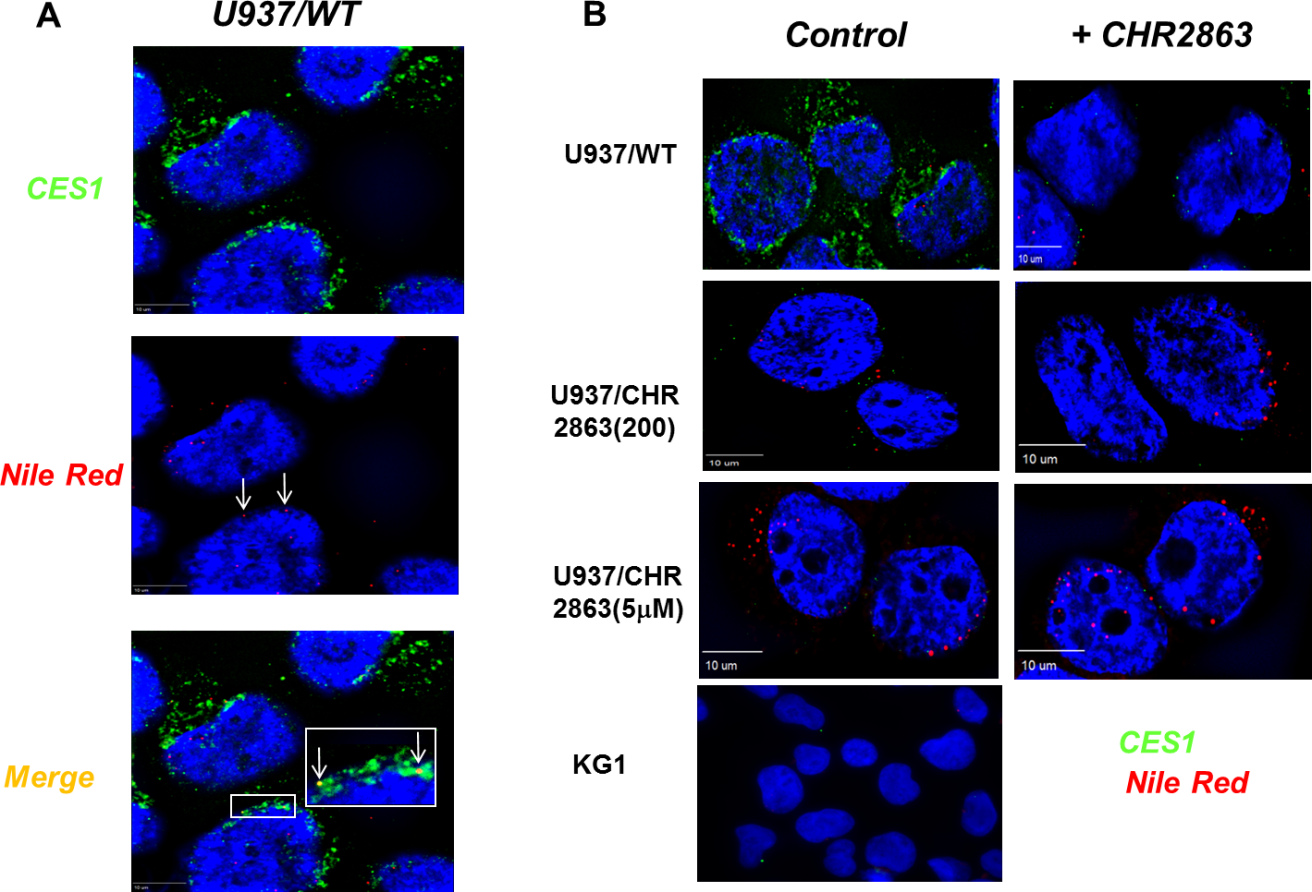
CES1 expression and identification of lipid droplets in U937/WT and CHR2863-resistant cells and CES1-lipid droplet co-localization **A.** Nile Red staining of lipid droplets and CES1 immunofluorescence detection by 3D digital imaging fluorescence microscopy. The inset depicts colocalization of CES1 with lipid droplets in U937/WT cells. **B.** Live cell 3D digital imaging microscopy of CES1 expression in U937/WT, U937/CHR2863(200), U937/CHR2863(5μM) cells and KG1 cells (as CES1-negative control). Left row represents control conditions, right row represent images after pulse exposure to CHR2863 (6 hr, 6 μM CHR2863).

Several studies have pointed out that the catalytic activity of CES1 is enhanced upon hydrophobic interactions with lipid droplets, which are cell organelles involved in neutral lipid (triglyceride and sterolester) storage [29-31]. Lipid droplets have also been reported in U937 cells and recognized for a role in cancer and inflammatory processes [32, 33]. Given this hydrophobic compartment association with CES1, lipid droplets may deserve consideration as a possible site of sequestration of non-metabolized hydrophobic CHR2863 as in U937/CHR2863(5μM) cells (Figure 2B). Staining of intracellular lipids within lipid bodies by Nile Red and visualization by 3D digital imaging fluorescence microscopy is shown in Figure 4A, demonstrating the variability in numbers and sizes of lipid droplets in U937/WT cells. Additionally, we examined whether CES1 co-localized with lipid droplets, which proved to be the case (Figure 4A, merged figure and inset).

The data for CES1 protein expression (Figure 3C) in U937/WT and CHR2863-resistant cells were further corroborated by live cell 3D digital imaging microscopy revealing a markedly decreased expression of CES1 in U937/CHR2863(200) cells and a dramatic loss of CES1 expression in U937/CHR2863(5μM) cells, comparable to those in CES1-negative KG1 cells [21] (Figure 4B). Moreover, 3D digital live cell imaging microscopy also showed a rapid decrease in CES1 immunoreactivity in parental U937/WT cells after 6hr pulse exposure to 6 μM CHR2863 (Figure 4B). Since this pulse exposure had no effect on CES1 protein levels analyzed by Western blotting (data not shown), this points to CHR2863-induced conformational alterations or post-translational modifications in CES1 which impair antibody binding to the enzyme in its native state.

### Increased number of lipid droplets in U937/CHR2863 cells

**Figure 5:**
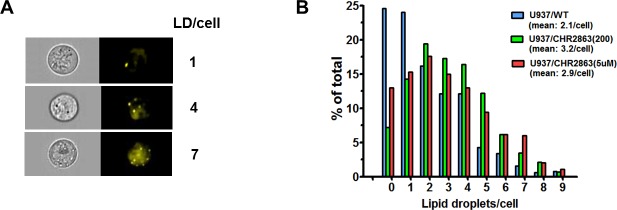
**A.** Lipid droplets staining in U937/WT cells with Nile Red and representative examples of image stream analysis sorting cells with 1, 4 or 7 lipid droplets per cell. **B.** Quantification of the distribution of lipid droplets in U937/WT, U937/CHR2863(200), and U937/CHR2863(5μM) cells by Nile Red staining and image stream analysis allowing assessment of numbers of lipid droplets per individual cell (as in (A)). Mean of two separate experiments performed in duplicate. All experiments included 0.06% DMSO solvent controls

Quantification of lipid droplet numbers in U937/WT and CHR2863-resistant cells was undertaken using live stream imaging analysis on Nile Red-stained cells with subsequent sorting of cells based on lipid droplet numbers per cell (Figure 5A). A representative distribution profile is depicted in Figure 5B, indicating that mean lipid droplet counts in U937/WT cells (2.1/cell) increased by 40% - 50% in U937/CHR2863(200) cells (3.2/ cell) and U937/CHR2863(5μM) cells (2.9/cell). The presence of lipid droplets in U937/WT and CHR2863-resistant cells was further confirmed by transmission electron microscopy (Supplementary Figure S4), which also indicated close physical contact of lipid droplets with mitochondria (Supplementary Figure S4C, subsection A8 and A9). These studies indicate that CHR2863 resistance is associated with increased lipid droplets content in CHR2863 resistant cells.

### Altered cholesterol homeostasis in CHR2863-resistant U937 cells

Beyond a pharmacologic function of CES1 in drug metabolism, the enzyme is also physiologically implicated in cholesterol homeostasis by regulating the hydrolysis of cholesteryl esters to free cholesterol [30, 34]. To explore whether the marked down- regulation of CES1 in U937/CHR2863(5μM) cells had an impact on cellular cholesteryl ester and free cholesterol content, these parameters were followed during 1-3 day cell growth (Supplementary Figure S5). After one day in culture, U937/CHR2863(5μM) cells had significantly higher levels of cholesteryl esters as compared to U937/WT cells. Upon prolonged cell growth, cholesteryl ester levels in U937/CHR2863(5μM) were normalized to those of U937/WT and U937/CHR2863(200) cells.

Collectively, these studies indicate that CHR2863 induces a differential response in CES1 and CES2 expression which, upon prolonged exposure, may contribute to acquisition of CHR2863 resistance and also impact cellular cholesterol homeostasis.

### Activation of Erk/Akt/mTOR pathway in U937/CHR2863 cells and marked gain rapamycin sensitivity

**Figure 6:**
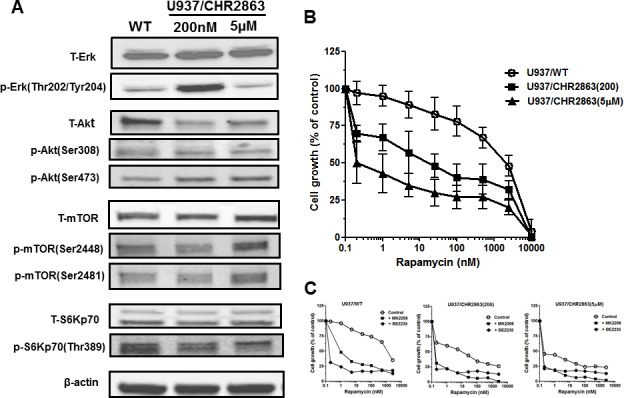
**A.** Expression levels of total and phosphorylated Erk, Akt, mTOR and S6K in U937/WT, U937/CHR2863(200) and U937/CHR2863(5μM) cells. **B.** Inhibition of cell growth by rapamcyin of U937/WT, U937/CHR2863(200) and U937/CHR2863(5μM) cells. Cell growth inhibition was assessed after 72 hrs drug exposure. Results represent the mean ± SD of 5-7 separate experiments. **C.** Inhibition of cell growth by rapamcyin of U937/WT, U937/CHR2863(200) and U937/CHR2863(5μM) cells upon co-incubation with non-toxic concentrations of the dual PI3K/mTOR inhibitor BEZ235 (10 nM) or Akt inhibitor MK2206 (100 nM). Cell growth inhibition was assessed after 72 hrs drug exposure and depicted as the mean of two separate experiments

Previous studies by Krige et al. [14] showed that the AP inhibitors induce an acute response of an intracellular amino acid deprivation leading to repression of mTOR activity as a master facilitator of protein synthesis [35]. We explored how CHR2863-resistant U937 cells overcame these initial responses by examining the phosphorylation status of mTOR and Akt as indicators of a pro-survival signal. Earlier experiments (not shown) demonstrated that CHR2863-resistant cells had similar levels of [^14^C]-arginine transport capacity as U937/WT cells and also did not show evidence (based on lack of LC3B cleavage) of autophagy induction as compensatory mechanism. Western blot analysis (Figure 6A) revealed increased ratios of phosphorylated Akt (Ser473) and phosphorylated mTOR over total Akt and mTOR in resistant cells compared to parental cells, pointing to the activation of the Akt/mTOR pathway in CHR2863-resistant cells. Akt activation was further indicated by a diminished sensitivity to the Akt inhibitor Perifosine (Supplementary Figure S6). A role for mTOR activation in CHR2863-resistant cells was confirmed by a marked gain in the sensitivity to rapamycin (Figure 6B); whereas U937/WT cells were relatively insensitive to growth inhibition by rapamycin (IC_50_: 2260 ± 960 nM), U937/CHR2863(200) cells displayed 54-fold increased sensitivity to rapamycin (IC_50_: 42 ± 38 nM). Rapamycin sensitivity was further enhanced (>1,000-fold) in U937/CHR2863(5μM) cells (IC_50_: 0.56 ± 0.41 nM). Notably, rapamycin sensitization in U937/WT cells could be induced by co-incubations with non-toxic concentrations of either the dual PI3K/mTOR inhibitor BEZ235 [36-39] or the Akt inhibitor MK2206 [40] (Figure 6C). Thus, conceivably, the marked gain in sensitivity to rapamycin may point to the common activation of Akt/mTOR pathway in myeloid leukemia cells [41]. Mechanistically, recent observations by Bar-Peled et al. [42] revealed that inactivating mutations in the DEPDC5 subunit of the GATOR1-mTOR inhibitory complex conferred mTOR hyperactivation and marked gain in sensitivity to rapamycin. We therefore examined whether DEPDC5 mutation could explain the marked gain of rapamycin sensitivity in CHR2863 resistant cells. However, no DEPDC5 mutations were identified in CHR2816-resistant cells (not shown). Together, these studies indicate that activation of Akt/mTOR can overcome the amino acid deprivation effects conveyed by AP-inhibitor drugs such as CHR2863.

**Figure 7:**
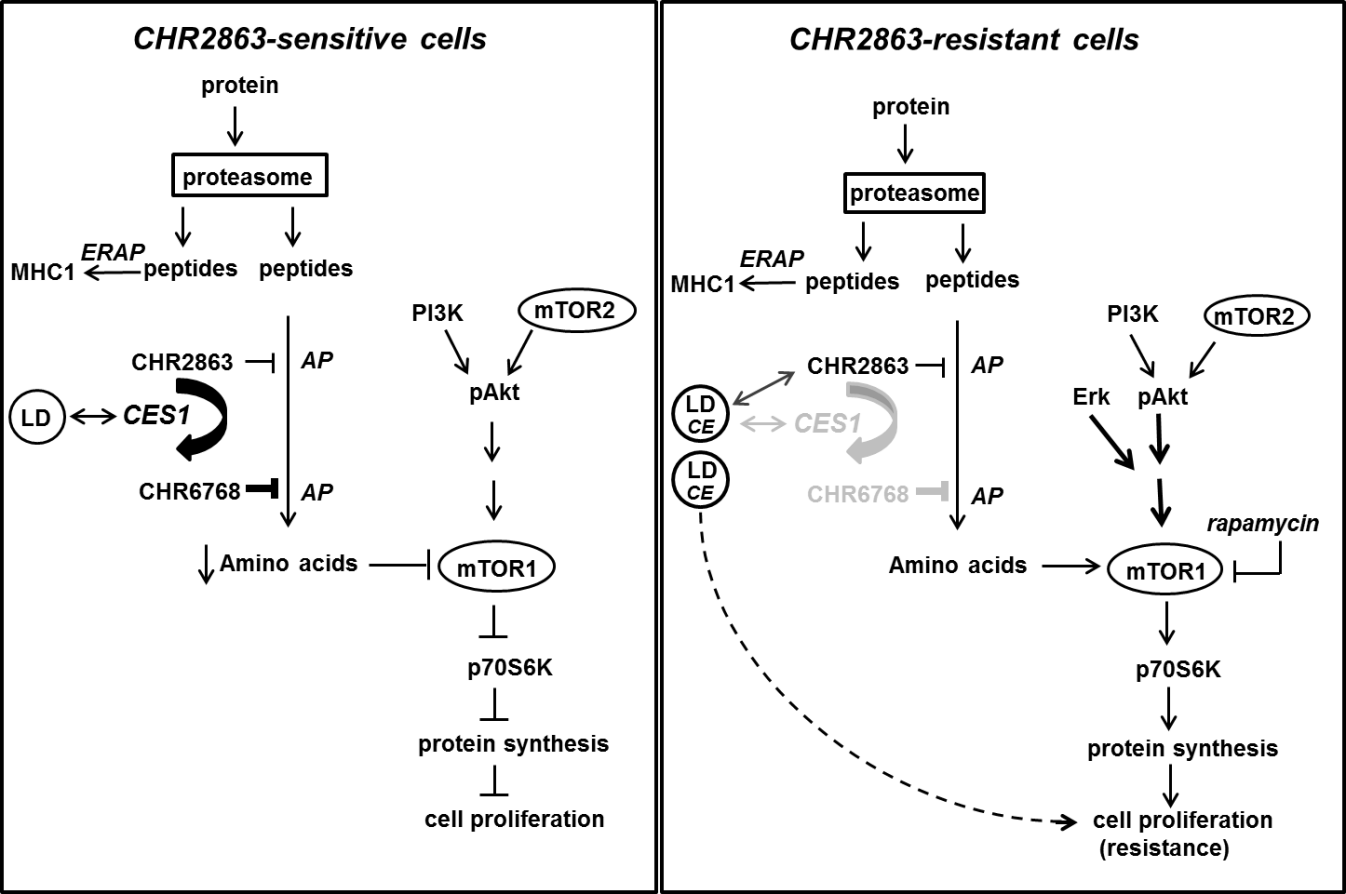
Composite model depicting mechanism of action of CHR2863 sensitivity in U937 cells (*left*) and mechanism of acquired resistance to CHR2863 in U937/CHR2863(5μM) cells (*right*) Parts of the (poly)peptides produced by proteasome-mediated protein degradation are processed for MHC class I presentation involving ERAP (ER-associated aminopeptidase). It is unknown whether CHR2863 or CHR6768 exert inhibitory effects on ERAP. Most polypeptides will be subject to full degradation to amino acids, involving aminopeptidase (AP) action, for renewed protein synthesis. Due to its hydrophobic nature, the cyclopentyl-ester conjugated compound CHR2863 can freely diffuse into cells and has potential to inhibit several APs [14]. The AP-inhibitory potency, however, is significantly improved upon conversion of CHR2863 to its acid metabolite CHR6768 which is accumulated and retained in cells. A likely candidate for CHR2863 conversion includes carboxylesterase 1 (CES1), which has a physiologic function in regulating cholesterol homeostasis, in particular in lipid droplet (LD) cell organelles. Conceivably, CES1 associated with LDs may provide a microenvironment that promotes CHR2863 conversion. CHR6768-induced inhibition of multiple APs will provoke an amino acid depletion which is sensed by mTOR leading to suppression of protein synthesis and inhibition of cell growth. In CHR2863-resistant cells, at least two adaptations took place. One involves a marked down-regulation of CES1 which may convey two effects; (i) rather than conversion, CHR2863 is sequestered in LD. Concomitantly, increased cholesteryl esters in LD may act as cell proliferation regulator, and (ii) loss of CHR6768-induced AP inhibition relieves part of the amino acid deprivation pressure, which could contribute to reactivation of mTOR activity. Second, mTOR reactivation may also be initiated separate from CES1 down-regulation (as in U937/CHR2863(200) cells) to promote protein synthesis and cell growth consistent with a resistant phenotype. This is achieved by Erk activation, as an early response in low resistant cells, and Akt/mTOR activation. Activation of mTOR in CHR2863-resistant cells is a targetable entity for its inhibitor rapamycin.

A composite summary model that accommodates all altered parameters contributing to the CHR2863 drug resistance phenotype is presented and discussed in Figure 7.

## DISCUSSION

Resistance modalities often disclose the Achilles heel of drug resistant cancer cells thereby offering a therapeutic avenue to overcome well defined chemoresistance phenotype [43-47]. The current study constitutes the first report aimed at unraveling the molecular basis of resistance to CHR2863. CHR2863 is an aminopeptidase inhibitor prodrug that structurally mimics the aminopeptidase inhibitor Tostedostat, which shows promising activities in AML treatment [15]. CHR2863 is a hydrophobic prodrug with an esterase motif rationally designed to be membrane permeable and is activated intracellularly via an esterase-dependent activity that converts it to the active hydrophilic congener CHR6768 which targets multiple aminopeptidases. Here, we demonstrate that acquired resistance to CHR2863 is a multifactorial mechanism including down-regulation of CES1 expression, impaired prodrug conversion with intracellular sequestration presumably in lipid droplets, and activation of the pro-survival ERK/Akt/mTOR pathway.

Carboxylesterases (CES) play a pivotal role in intracellular metabolic processes of drug detoxification or bioactivation as well as lipid/cholesterol homeostasis [26, 27, 30]. The role of CES1 in CHR2863 resistance was most evident in the highly resistant U937/CHR2863(5μM) cells where both a marked down-regulation of CES1 mRNA and CES1 protein were associated with impaired prodrug conversion. However, for low level CHR2863 resistant U937/CHR2863(200) cells, down-regulation of CES1 mRNA was not accompanied by similarly reduced CES1 protein levels, albeit a ≈ 25% reduction was observed in conversion of CHR2863 to CHR6768 compared to U937/WT cells. This suggests that additional factors contribute to regulation of CES1 protein and catalytic activity at low resistance levels versus highly resistant cells. In this respect, it should be taken into account that within a window of selective concentrations of 0.2 to 5μM, even CHR2863 as a prodrug, in analogy with CHR2797 [14], would impose increasing inhibitory pressure on multiple APs, thus calling for additional modalities to neutralize these deleterious effects. In macrophages, redistribution of CES1 from the cytoplasm to lipid droplets has been reported in response to lipid loading [48]. It is noteworthy that in response to down-regulation of CES1, CES2 was upregulated, probably as a compensatory mechanism. Since upregulation of CES2 had no pharmacologic impact on CHR2863 conversion in U937/CHR2863(5μM) cells, this would imply that CHR2863 is a poor substrate for CES2 and cannot facilitate sufficient conversion to CHR2863 to compensate for CES1 down-regulation. Rather than displaying pharmacological functions, CES2 upregulation may be a physiologic compensatory mechanism preserving some essential cellular functions, in particular in relation to cholesterol homeostasis, i.e. hydrolysis of cholesteryl esters to free cholesterol. Studies by Zhao et al. [49] showed that stable over expression of CES1 in human macrophage THP1 cells promoted the extrusion of free cholesterol. Conversely, pharmacologic inhibition of CES1 induced cholesteryl ester retention [34], whereas shRNA-dependent CES1 knockdown in THP1 cells was accompanied by compensatory CES3 upregulation to sustain cholesteryl ester hydrolytic activity [50]. Moreover, the same study demonstrated that CES3 transfection decreased the numbers of lipid droplets in THP1 cells. Our data (including Supplementary Figure S5) are consistent with these observations, except that upregulation of CES2 rather than CES3 in U937/CHR2863 cells may serve as a compensatory mechanism in cholesterol homeostasis. In this context recent studies pointed to the role of increased cholesterol ester levels as growth regulators in leukemia cells [51]. Whether increased cholesterol ester levels in CHR2863-resistant cells indeed contribute to the resistant phenotype deserves further studies. Lastly, EM studies indicating that in CHR2863-resistant cells lipid droplets had physical contacts with mitochondria (Suppl. Fig S4B) implying that lipolytic activity within lipid droplets and the release of free fatty acid for β-oxidation in mitochondria holds relevance for energy transfer [52].

Direct evidence for CHR2863 sequestration in subcellular compartments such as lipid droplets is limited by subcellular fractionation techniques that would retain low quantities of CHR2863 hydrophobic prodrug per cell (Figure 2B). Nonetheless, several lines of indirect evidence point to the plausible role of lipid droplets in the CHR2863 resistance phenotype. Conceivably, given the dominant hydrophobicity of CHR2863, CES1 surrounding lipid droplets would provide a more optimal microenvironment for interaction and hydrolysis of this CHR2863 prodrug than the water soluble cytoplasm [30]. Down-regulation of CES1 expression and impaired hydrolysis of CHR2863 would then drive its marked accumulation and sequestration in lipid droplets. In analogy, CHR2863 induced swelling of malaria digestive vacuoles [53], which are known to harbor lipid droplets [54;55]. Apart from their established role in cholesterol ester storage, lipid droplets have also been recognized as sites of arachidonic acid metabolism leading to the production of leukotrienes and prostaglandins, both mediating inflammatory processes [32, 56]. In this regard, it is worthwhile to note that the active metabolite of Tosedostat displayed potent inhibitory effect on leukotriene A4 hydrolase activity (IC_50_: >10,000 nM for prodrug vs 8 nM for the active metabolite). Indications that the development of CHR2863-resistance was accompanied by alterations in arachidonic acid metabolism could be consistent with observations that U937/CHR2863 cells upregulated the expression of the MDR efflux transporter MRP4 (Suppl. Fig S2), the function of which has been reported in intracellular vesicles of U937 cells as a facilitator of leukotreine B4, leukotriene C4 [57] as well as prostaglandin E2 [58] extrusion. These considerations come on top of other functions of lipid droplets unrelated to lipid metabolism [31, 59-61], including protein trafficking, temporary sequestration of proteins and handling proteins prone for destruction, the latter of which may be of relevance in CHR2863 targeting the proteasome/ aminopeptidase pathway of protein degradation. In these functions, lipid droplets act in a dynamic fashion by organizing transient association with other cellular organelles as endoplasmic reticulum, mitochondria, endosomes as well as the cytoskeleton.

Studies by Krige et al. [14] showed that although CHR2797 (Tosedostat) prodrug conversion is a critical step in exerting its pharmacological effect, equally important is whether or not target cells had the capacity to overcome the induction of an amino acid deprivation response and consequent suppression of mTOR activity [62]. In other words, cells with efficient prodrug conversion but a proficient amino acid deprivation response, displayed reduced drug sensitivities. This condition may be mimicked by U937/CHR2863(200) cells which had 70% residual conversion of CHR2863 to CHR6768 compared to parental U937/WT cells, but acquired a low level of CHR2863 resistance due to reactivation of Akt/mTOR. Beyond Akt/mTOR, in U937/CHR2863(200) cells ERK activation was also noted as a transient early pro-survival response, which was not retained in U937/CHR2863(5uM) cells. Akt/mTOR activation in combination with impaired CHR2863 prodrug conversion elicited a high level of resistance in U937/CHR2863(5μM) cells. It is intriguing to note that these phenotypes could be efficiently monitored by assessment of sensitivity to the mTOR inhibitor rapamycin [63, 64]. Whereas U937/WT cells were relatively insensitive to rapamycin [65], U937/CHR2863(200) cells and U937/CHR2863(5μM) cells already gained a substantial increase in rapamycin-sensitivity at clinically relevant concentrations of 10 nM; this novel finding has major therapeutic implications for the overcoming of CHR2863 resistance in the clinical setting. Mechanistically, the increased sensitivity to inhibition of an overactivated pathway such as Akt/mTOR is compatible with the ‘dam model’ as proposed for overactivated pro-survival pathways such as BCR-ABL fusion [66].** This upstream survival anti-apoptotic mechanism has no downstream anti-apoptotic backing. Hence, upon pharmacologic inhibition of the overactivated and uncontrolled dam kinase like BCR-ABL, cells become extremely sensitized and hence collapse and die as the dam is gone.

Collectively, a multifactorial mechanism appears to underlie acquired resistance to CHR2863 that includes loss of CES1 expression, lack of prodrug conversion, drug sequestration as well as Akt/mTOR activation.

It is a recurrent theme whether mechanisms of drug resistance observed in model systems will also be operative in a clinical setting. For the treatment of myeloid leukemia, CHR2797 (Tosedostat) is not administered as single agent but usually in combination with other chemotherapeutics, i.e. daunorubicin and cytarabine [15], for which CHR2863-resistant U937 myeloid cells retained activity (Table 1). Interestingly, the current study raised some additional potential combinations that may merit further exploration. One would be a combination of CHR2863 with CPT-11/irinotecan which showed collateral sensitivity in CHR2863-resistant U937 cells (Table 1). This is likely attributable to the increased expression of CES2 which facilitates activation of CPT-11 [27;28, 67, 68]. This combination may not only be effective in CHR2863-resistant myeloid cells, but to previously unexposed cells as CES2 upregulation was also noted after short term CHR2863 exposure (Suppl. Figure S3A/B). Notably, the role of CES2 in collateral sensitivity to CPT-11 in CHR2863-resistant cells was confirmed by the fact that pharmacologic inhibitors of CES2, e.g. loperamide and benzil [69] abrogated this sensitizing effect (not shown). The opposite response in CES1 and CES2 expression upon CHR2863 exposure may neutralize a potential combination effects for capecitabine as this 5-FU prodrug can be activated by both esterases [70, 71]. Last but not least, the dramatic gain of sensitivity to rapamycin in U937/CHR2863 cells strongly calls for further examination of combinations of aminopeptidase inhibitor (pro)drugs and rapamycin or other rapalogs. As such, the expanded knowledge of mechanisms underlying loss of efficacy to aminopeptidase inhibitors may guide more rationalized applications of this type of drugs as single agent or in combination therapies, in order to achieve improved therapeutic targeting of monocytes/macrophages in either a cancer or (chronic) inflammatory disease setting.

## MATERIALS AND METHODS

### Chemicals

The compounds CHR2863; (6S)-[(R)-2-((S)-Hydroxy-hydroxycarbamoyl-methoxy-methyl)-4-methyl-pentanoylamino]-3,3 dimethyl-butyric acid cyclopentyl ester, CHR6768; (6S)-[(R)-2-((S)-Hydroxy-hydroxycarbamoyl-methoxy-methyl)-4-methyl-pentanoylamino]-3,3 dimethyl-butyric acid, CHR5346; (6S)-[(R)-2-((S)-Hydroxy-hydroxycarbamoyl-methyl)-4-methyl-pentanoylamino]-3,3 dimethyl-butanoic acid cyclopentyl ester; non-cleavable ester, CHR2875; (S)-[3-(7-Hydroxycarbamoyl-heptanoylamino)-benzylamino-phenyl acetic acid cyclopentyl ester, and CHR2880 ((S)-[3-(7-Hydroxycarbamoyl-heptanoylamino)-benzylamino-phenyl acetic acid) were synthesized by Chroma Therapeutics UK [14;21] and dissolved in dimethylsulfoxide as 10 mM stock solutions and stored at −20°C.

CPT-11/Irinotecan was obtained from Tocris Biosciences (Ellisville, MO, USA), Bortezomib from Millennium Pharmaceuticals (Cambridge MA, USA), MTX from Pharmachemie (Haarlem, The Netherlands), Carfilzomib from Onyx Pharmaceuticals (South San Francisco, USA), Rapamycin (JS Research Chemicals Trading, Wedel, Germany) and MK571 from Enzo Life Sciences (Antwerp, Belgium). Other drugs, including bestatin, daunorubicin, cytarabine (Ara-C), capecitabine/Xeloda (5′-deoxyfluorouridine), methyl-β-cyclo-dextrin, loperamide, benzil and Nile Red were purchased from Sigma-Aldrich (St. Louis, MO, USA). Triton-X100 and paraformaldehyde were from Merck (Darmstadt, Germany). Perifosine was a gift from AeternaZentaris (Frankfurt, Germany). MK2206 and NVP-BEZ235 were obtained from Selleckchem (Europe).

### Antibodies

The following antibodies were used: CES1 (a polyclonal antibody from Proteintech Group, Chicago, IL, USA, 16912-1-AP, and a monoclonal antibody from Lifespan Biosciences, Seattle, WA, USA, LS-C498701, both 1:1000 dilution), CES2 (Santa Cruz Biotechnology, Santa Cruz, CA, USA, Sc-100685, 1:250 dilution, and Life Span Bio, clone 4F12, LS-B6190, 1:500 dilution), CES3 (1:1000, Protein Europa, 14587-1-AP, 1:1000 dilution) and MRP1 (MRPr1, 1:500), MRP2 (M2 III-6, 1:500), MRP3 (M3 II-21, 1:500), MRP4 (M4 I-10, 1:250), MRP5 (M5 I-10, 1:250), Pgp (JSB1, 1:500), BCRP (BXP53, 1:200) as described before [72]. The following poly/monoclonal rabbit antibodies were all from Cell Signalling Technology (Danvers, MA, USA) at a 1:1000 dilution: Total Akt (#9272), phospo-Akt (Ser308) (C31E5E) (#2965), phospho-Akt (Ser473) (#9271), total mTOR (7C10) (#2983), phospho-mTOR (Ser2448) (#2971), phospho-mTOR (Ser2481) (#2974), total S6 kinase (#9205), phospho-S6 kinase (Thr389) (#9205), P44/42 MAPK (Erk1/2) (3A7) (#9107S) and phospho-p44/42 MAPK (T202/Y204) (pErk1/2) (#9101L). β-Actin antibody was from Sigma-Aldrich, St. Louis, MO, USA, A2172, 1:10,000). Secondary antibodies included goat anti-mouse or goat anti-rabbit antibodies conjugated to IRDye®800CW (1:10.000, Odyssey; LI-COR, Biosciences, Nebraska, USA); rabbit anti-rat/HRP, rabbit anti-mouse/HRP (1:2000, DAKO, Glostrup, Denmark) or goat anti-rabbit/HRP (1:2000, Santa Cruz Biotechnology, CA, USA) and goat anti-rabbit Alexa 633 (Life Technologies, Paisley, UK).

### Clinical acute myeloid leukemia samples

Stored cryopreserved pre-treatment samples from AML patients (peripheral blood and/or bone marrow) were collected according to Helsinki protocol. Samples were classified according to the FAB system and included the following subtypes; M0 (*n* = 2), M1 (*n* = 2), M2 (*n* = 3), M4 (*n* = 2), and M5 (*n* = 5). Peripheral blood was obtained from 3 healthy volunteers with written informed consent.

### Cell culture and development of CHR2863 resistance

The human myelomonocytic leukemia cell line U937 (ATCC, Manassas, VA, USA) was grown in RPMI-1640 culture medium (Lonza, Verviers, Belgium) supplemented with 5% fetal calf serum (FCS, PAA Cell Culture Company, Pasching, Austria), 20 mM HEPES, 2 mM L-glutamine, and 100 U/ml penicillin/streptomycin (all from Lonza, Verviers, Belgium). Cells were cultured in 25cm^2^ culture flasks (Greiner Bio-One GmbH, Frickenhansen, Germany) in 10 ml medium at an initial density of 3 × 10^5^ cells/ml and in a humidified atmosphere at 37°C and 5% CO_2_. Cell cultures were refreshed every 3-4 days.

Acquired resistance to CHR2863 was induced by exposing U937/WT cells to a starting concentration of 15 nM CHR2863 (IC_10_) for one week. Then, the concentration of CHR2863 was gradually stepwise increased when cells had adapted to drug increments by exhibiting cell growth comparable to control U937/WT cells. Over the course of CHR2863 increments, two sublines of CHR2863 resistant U937 cells were selected for further detailed characterization; (a) one with a relatively low level of acquired resistance (≈ 14-fold) isolated after 2.5 months when grown in the presence of 200 nM CHR2863 (further designated as U937/CHR2863(200), and (b) another with a high level of CHR2863 resistance (> 250-fold) isolated after 5-6 months when grown in the presence of 5 μM CHR2863 (further designated as U937/CHR2863(5μM) cells).

### Cell growth inhibition assay

Growth inhibition assays on U937/WT cells and CHR2863 resistant sublines were performed essentially as described previously [24]. In short, 0.5 ml cell suspensions were plated in 48-well plates at an initial density of 1.25 × 10^5^ cells/ml. An untreated control and 7 different drug concentrations (covering 2 log concentrations) were included in each experiment. As vehicle control, maximal concentrations of 0.06% DMSO were included. Cells were grown in a humidified atmosphere at 37°C and 5% CO_2_ and after 72 hours drug exposure, cell counts were performed with hemocytometer and cell viability was checked by trypan blue exclusion.

### RNA isolation, cDNA synthesis and qRT-PCR CES1 & CES2, CES1 siRNA

Extraction of total RNA from 1-2 × 10^6^ cells was performed with Trizol (Invitrogen, Paisley, UK) according to manufacturers. For cDNA synthesis, the DyNAmo cDNA Synthesis Kit (Thermo Scientific, Waltham, MA, USA) was used as follows: 500 ng RNA were diluted in RNase-free water to a total volume of 7 μl and mixed with 10 μl RT buffer (containing 10 mM MgCl_2_ and a dNTP mix), 1 μl of random hexamers (300 ng/μl) and 2 μl of M-MuLV RNase H+ reverse transcriptase (RT). PCR was performed under the following conditions: 10 min at 25°C for primer extension, 30 min at 37°C for cDNA synthesis and 5 min at 85°C to terminate the reaction (inactivation of M-MuLV). The samples were stored at −20°C until use.

qRT-PCR was performed to determine the mRNA expression levels of CES1 and CES2. cDNA was diluted 1:10 by adding 180 μl of RNase-free water to the sample volume of 20μl. For a duplicate reaction, 29.5 μl of the TaqMan Universal PCR Master Mix (Applied Biosystems, Foster City, CA, USA), 15.5 μl RNase-free water and 2.5 μl probed primers (TaqMan Gene Expression Assays Hs00275607_m1 for CES1, Hs00187279_m1 for CES2 and Human ACTB Endogenous Control (VIC®-MGB, probe # 4326315E for β-actin, all from Applied Biosystems) were mixed with 12.5 μl of diluted cDNA. 25 μl of each mix were transferred in duplicate to a 96-well PCR plate. The qRT-PCR was performed with the Applied Biosystems 7500HT sequence detection system apparatus utilizing optimal primer concentrations, i.e., associated with minimum standard deviations between CT values. A validation experiment was performed to demonstrate that the efficiencies of the target (CES1 and CES2) and reference (β-actin) gene amplifications were approximately equal, using a standard curve method with several dilutions (from 1:10 to 1:10000) of a cDNA sample from untreated control cells. The results were analyzed using the ΔΔCt method, where Ct values are normalized to the reference gene (β-actin) and shown relative to a control. Relative mRNA expression levels are depicted as 2^(−ΔΔCt)^, i.e. 2^(-Ct target - Ct β-actin - Ct control)^.

Knockdown of CES1 expression was performed using Silencer Select pre-designed siRNA (Applied Biosystems, siRNA ID S2921), sense: CCAUGGAGCUUUGUGAAGAtt, antisense: UCUUCACAAAGCUCCAUGGtt according to manufacturers’ procedures. In short, U937/WT cells were resuspended at 20°C in serum-free RPMI medium at an initial density of 4 × 10^5^ cells/ml. A solution of CES1 siRNA (25 pmol in nuclease-free water) or Stealth RNAi negative control (Invitrogen, Carlsbad, CA, USA) were preincubated with lipofectamine (Invitrogen) for 10 min at room temperature and then added to the cells. After 4 hours incubation, 10% FCS was added to each flask and after 24 hours, cells were collected and analyzed for CES1 knockdown efficiency by qRT-PCR as described above.

### Western blotting

Cells (3 × 10^6^) were harvested in the mid-log phase of growth, washed 3 times with ice-cold PBS after which cell lysates were prepared by resuspending in 150 μl lysis buffer (Cell Signaling Technology, #9803) containing 4% PIC (Protease Inhibitor Cocktail) and 1 mM NaVO_4_. After centrifugation (13,000 x g for 10 min), protein content in the supernatant fraction was determined using the Bio-Rad Protein Assay (Munich, Germany). Protein aliquots (30 μg) of cell lysates were fractionated on a 4-20% TGX pre-cast SDS PAGE gel (BioRad) and next transferred onto a polyvinylidene difluoride (PVDF) membrane (Millipore, Billerica, MA, USA) suitable for the Odyssey Infrared Imaging System (PerkinElmer, Zaventem, Belgium) for chemoluminescent detection. The membranes were pre-incubated for 1 hour in blocking buffer (Odyssey Blocking Buffer, LI-COR, Biosciences) or PBST (PBS and 0.05% Tween20, Merck, Darmstadt, Germany) containing 5% non-fat dry milk (Biorad, Munich, Germany). After blocking, the membranes were incubated overnight at 4°C with specific primary antibodies. β-Actin was used as the control for equal loading. After 3 washing steps with PBS/0.05% Tween20 (Merck, Germany), the membranes were incubated for 1 hour with appropriate secondary antibodies. Detection of antibody binding was obtained using the LI-COR Odyssey scanner (Biosciences) according to the manufacturers’ instructions, or incubated for 5 min with the ECL Plus detection solution (GE Healthcare, Buckinghamshire, UK) and exposed to an Amersham high performance chemoluminescence film (GE Healthcare, Buckinghamshire, UK). Digital image acquisition and quantification was performed using the Odyssey infrared imaging system software (version 3.0.16, LI-COR Biosciences, Nebraska, USA)

### LC-MS/MS analysis of CHR2863 prodrug conversion to its acid metabolite CHR6768

Intracellular conversion of CHR2863 to its acid form and active metabolite CHR6768 was analyzed by incubating 1.5 × 10^6^ U937/WT and U937/CHR2863 cells (in the absence of selecting drug) in a 25 cm^2^ tissue culture flask in 5 ml RPMI-1640 medium/5% FCS at 37°C in a humidified 5% CO_2_ atmosphere. After 24 hours, cells were exposed for 6 hours to 6 μM CHR2863 (unless otherwise indicated). Cells were then centrifuged and 500 μl of supernatant conditioned medium collected and stored at −80°C. Cells were then washed twice with 7.5 ml ice-cold PBS, counted and frozen at −80°C for analysis. A similar procedure was followed for the assessment of the conversion of the HDAC-inhibitor prodrug CHR2875 to CHR2880 [21]. CHR2863 and CHR2875 and their primary active metabolites were determined by a validated liquid chromatography tandem mass spectrometric assay, essentially as described by Krige et al. [14]. Briefly, frozen cell pellets were allowed to warm to room temperature before being re-suspended in minimal volume of purified water. The subsequent homogenous suspension was diluted with water to achieve 5.0 × 10^6^ cells/ml, prior to being snap frozen in liquid nitrogen. Following re-thawing 100 μl of the homogenized cell suspension was extracted with 40 μl of acetonitrile containing 250 μg/ml BB-1090 (internal standard). After 10 minutes ultra-sonication and centrifugation (for 2 min, at 10 000 g) 50 μl of the supernatant was transferred to a 96-well plate for LCMS/MS analysis.

Optimized liquid chromatography was performed on a 4 μm C18 Hyperclone column (Phenomenex, 50 × 2 mm) with gradient elution over 4 min at a flow rate of 250 μl/min. Initial conditions consisted of 85% A (aqueous 0.1% formic acid) and 15 % B (0.1% formic acid in acetonitrile), changing to 15% A: 85% B after 1 min and subsequently holding for a further 3 min. Mass spectroscopic detection was performed at optimized conditions as follows; Ion spray voltage - 3800 volt, Capillary temperature 420°C, nebulizer gas - 10 litres per min, auxiliary gas - 6 litres per min. Detection parameters for each mass were optimized with mobile phase B for CHR2863 (421.1 / 260.1), CHR6768 (353.160 / 260.1) CHR2875 (496.0 / 382.2), CHR2880 (428.0 / 382.2) and BB-1090 (366.2 / 119.7). Data quantification was performed with Analyst (AB sciex B.V) ver 5.2 in combination with Dionex Mass Link chromatography software ver 2.10.

### Image stream analysis for lipid droplet counts

Cells (2 × 10^6^) in the mid-log phase of growth were harvested and washed 3 times with PBS/0.1% BSA and resuspended in 1 ml PBS/0.1% BSA. Cells were then incubated in the dark for 4 min at 25°C with Nile Red (0.1 μg/ml final concentration from stock solution of 1 mg/ml in 10% acetone). Cells were then washed 3 times protected from light with ice-cold PBS/0.1% BSA and processed by ImageStream ISX analysis (Amnis, Seattle, WA, USA), an instrument that combines microscopy and flow cytometry in one platform. By imaging cells in flow, the system - on a per cell basis - allows the measurement of brightness, size and localization of fluorescently labeled subcellular components and compilation of these data into the population statistics of conventional FACS analysis. The analysis software (Amnis, Seattle) utilizes two tool sets; “masks” giving location to an object in an image and “features” enabling measurement of the physical properties of the objects defined by the mask. Thus, Nile Red labeled lipid droplets in a cell were masked as objects with a user defined pixel radius and brightness. The spot count feature then counted the objects that met these criteria. The data is displayed as the percentage of cells in a given sample with a specified number of spots from a minimum of 10,000 cells analyzed.

### 3D digital imaging fluorescence microscopy

Cytospins were prepared from U937/WT, U937/CHR2863(200) and U937/CHR2863(5μM) cells grown for 2 days in absence of selecting drugs. Cells were then fixed with 4% paraformaldehyde in PBS for 10 min at room temperature. After washing the slides with PBS, cells were permeabilized with PBS/0.1% Triton-X100 for 10 min at 4°C. After washing with PBS, the cells were blocked in 10% FCS for 30 min at room temperature. Next, cells were washed three times in 100 μl PBS and then incubated with primary CES1 polyclonal antibody (1:400, diluted in PBS/0.1%BSA) and incubated for 1 hour at room temperature. Cells were then washed three times with PBS and incubated with secondary antibody (goat anti-rabbit Alexa 633, 1:300 diluted in PBS/0.1%BSA) for 45 min at room temperature. After three times washing, cells were stained with Nile Red (1:2500 dilutions from stock solution of 1 mg/ml in 10% acetone in PBS) for 5 min. After washing 3 times with PBS, cells were air dried in a flow chamber for 1min followed by DAPI staining. The cytospin preparation was then covered by a cover slip, fixed with nail polish, and prepared for microscopy analysis. To this end, fixed cells were examined with a Zeiss Axiovert 200M Marianas™ inverted microscope, equipped with a motorized stage (stepper-motor z-axis increments: 0.1 μm), and a turret of four epifluorescence cubes (FITC, Cy-5, Cy-3, AMCA as well as a DIC bright field cube). A cooled CCD camera (Cooke Sensicam SVGA [Cooke Co., Tonawanda, NY], 1,280 × 1,024 pixels) recorded images with true 16-bit capability. The camera is linear over its full dynamic range (up to intensities of over 4,000) while dark/background currents (estimated by the intensity outside the cells) is typically < 100. Exposures, objective, montage, and pixel binning were automatically recorded with each image stored in memory (Dell Dimension workstation: Quad-core processor, 16GB RAM). The microscope, camera, and data processing were controlled by SlideBook™ software (SlideBook™ version 5.5.2.0 [Intelligent Imaging Innovations, Denver, CO]). All microscopy was performed with a custom 40X or 63X oil-immersion lens (Zeiss). The motorized filter cubes allowed acquisition of one composite image (on all four different fluorescent wavelengths) within 2 s. The data acquisition protocol included optical planes to obtain 3-D definition. Moreover, the software used is fully equipped to acquire, process (several deconvolution modes), and display true 3-D data and was used throughout the experiments.

### Electron microscopy

Cells in the mid-log phase of growth were fixed using an overnight incubation in 2% (vol/vol) glutaraldehyde in phosphate buffer for 30-minutes and 1.5% (wt/vol) osmium tetroxide for 10 min, dehydrated with acetone, and embedded in Epon812. Ultrathin sections (60-70 nm) were collected on 300-mesh Formavar-coated nickel grids. The sections were counterstained with 2% uranyl acetate and lead citrate and were examined in a Jeol 1200EX electron microscope. Photographs were finally printed using a Leitz Focomat IIc.

### Cholesterol assay

Intracellular levels of cholesterol and cholesterylesters were determined by the Amplex Red fluorometric assay (Molecular Probes, Eugene, OR, USA) according to the manufacturers’ procedure and described by Li et al. [73]. Briefly, 1 × 10^6^ cells were harvested at days 1, 2 and 3 after plating of regular cell cultures of U937/WT cells, U937/CHR2863(200) and U937/CHR2863(5uM) cells at their selective concentrations of CHR2863 in the growth medium. Cells were washed 3 times with ice-cold PBS and aliquots of 2 × 10^5^ cells were centrifuged in Eppendorf tubes and cell pellets were stored at −20°C until analysis. Levels of total cholesterol (free-cholesterol and cholesteryl esters) were expressed as ng/10^6^ cells. As a control, cholesterol levels were analyzed in U937/WT cells incubated with 1 mM of the cholesterol-depleting agent methyl-β-cyclo-dextrin (MβCD).

### Statistics

For comparison between groups, a two-sided paired Student's t-test was used. Differences were considered to be significant at *p* < 0.05.

## SUPPLEMENTARY MATERIAL FIGURES



## Abbreviations

CHR2863: ((6S)-[(R)-2-((S)-Hydroxy-hydroxycarbamoyl-methoxy-methyl)-4-methyl-pentanoylamino]-3,3 dimethyl-butyric acid cyclopentyl ester)
CHR6768: ((6S)-[(R)-2-((S)-Hydroxy-hydroxycarbamoyl-methoxy-methyl)-4-methyl-pentanoylamino]-3,3 dimethyl-butyric acid
CHR5346: ((6S)-[(R)-2-((S)-Hydroxy-hydroxycarbamoyl-methyl)-4-methyl-pentanoylamino]-3,3 dimethyl-butanoic acid cyclopentyl ester; non-cleavable ester
CHR2875: ((S)-[3-(7-Hydroxycarbamoyl-heptanoylamino)-benzylamino-phenyl acetic acid cyclopentyl ester)
CHR2880: (S)-[3-(7-Hydroxycarbamoyl-heptanoylamino)-benzylamino-phenyl acetic acid)
