# Supplementary material for: Multifactorial resistance to aminopeptidase inhibitor prodrug CHR2863 in myeloid leukemia cells: down-regulation of carboxylesterase 1, drug sequestration in lipid droplets and pro-survival activation ERK/Akt/mTOR
Source: Oncotarget. 2015 Oct 19;7(5):5240–57. doi: 10.18632/oncotarget.6169 (PMC4868683; doi:10.18632/oncotarget.6169)
Supplement: Supplementary file 1 [file oncotarget-07-5240-s001.pdf]

Multifactorial resistance to aminopeptidase inhibitor prodrug CHR2863 in myeloid leukemia cells: down-regulation of carboxylesterase 1, drug sequestration in lipid droplets and pro-survival activation ERK/Akt/mTOR

Supplementary Material

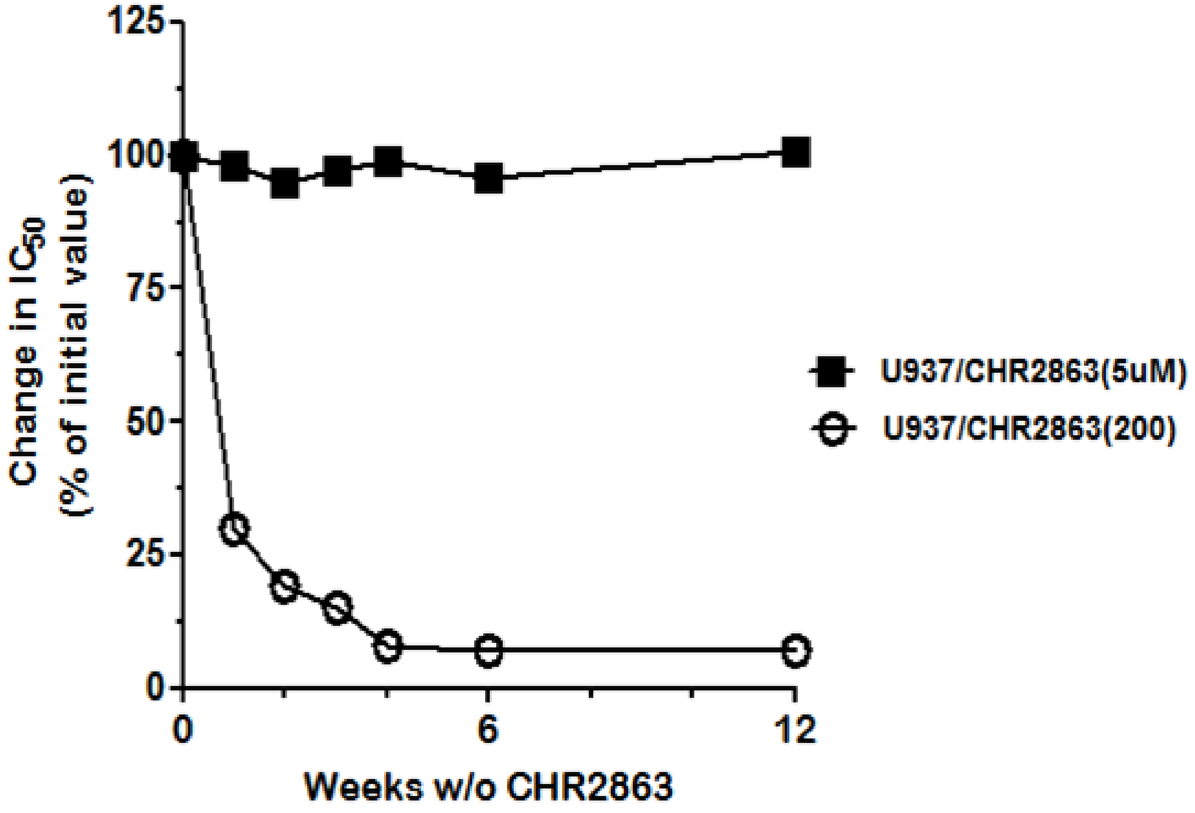

Figure S1: Stability of CHR2863 resistance in U937/CHR2863(200) and U937/CHR2863(5uM) cells. U937/CHR2863(200) and U937/CHR2863(5uM) cells were transferred to CHR2863-free medium and at selected time interval over a period of 3 months, cells were examined for their growth inhibition by CHR2863. IC50 values for 50% growth inhibition at the selected time points we depicted as relative to t=0 at the start of transfer in drug-free medium.

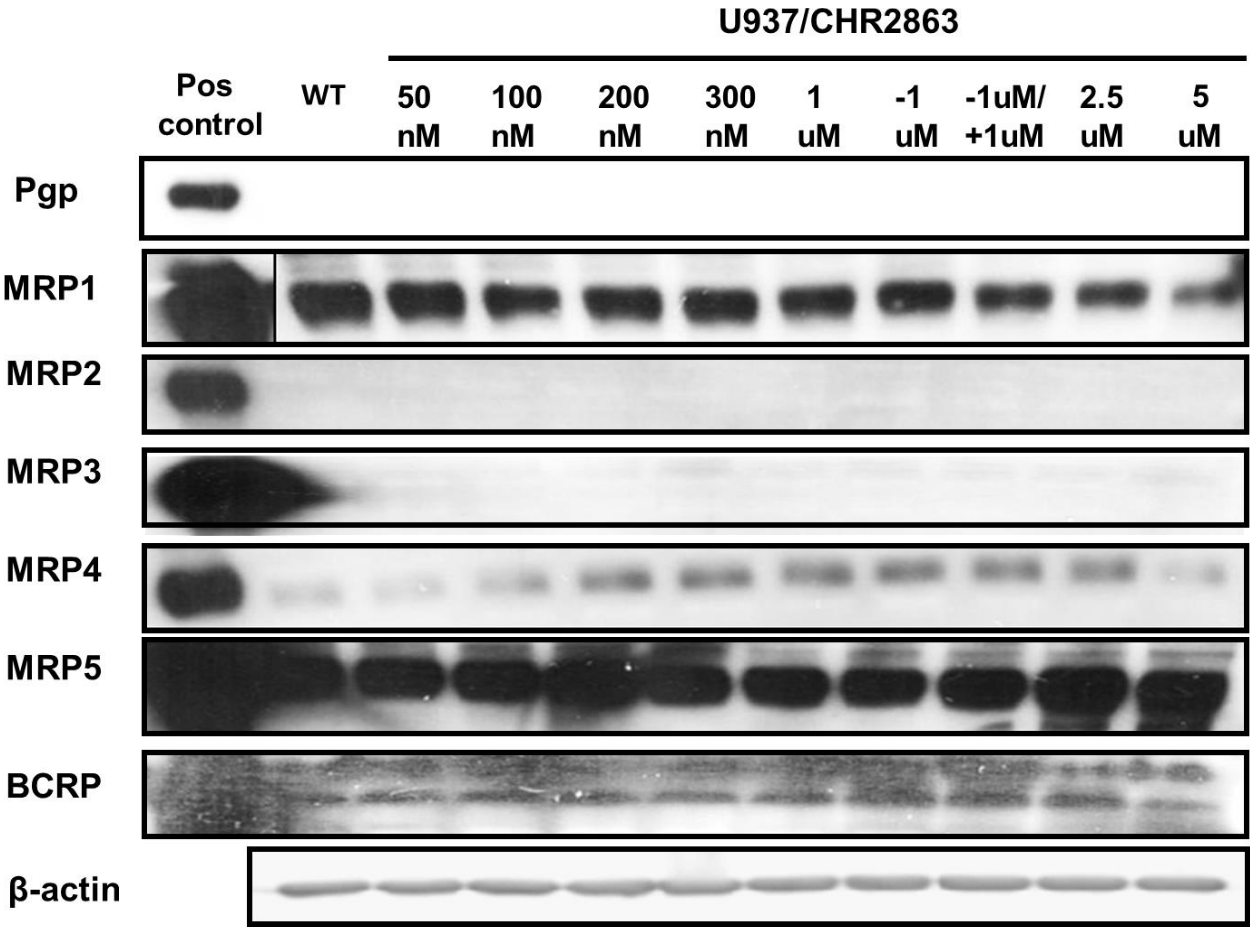

Figure S2: Expression levels of various established drug efflux transporters of the ATP-Binding Cassette family in U937/WT cells and U937 cells selected for growth in the presence of 50 nM to 5µM CHR2863. U937/CHR2863(-1 µM) refers to U937/CHR2863(1 µM) grown without CHR2863 for 2 weeks, whereas U937/CHR2863(-1µM/+1µM) after rechallenge with 1 µM CHR2863 for 2 weeks. Positive controls included previously reported cell lines overexpressing each of these transporters [24;72].

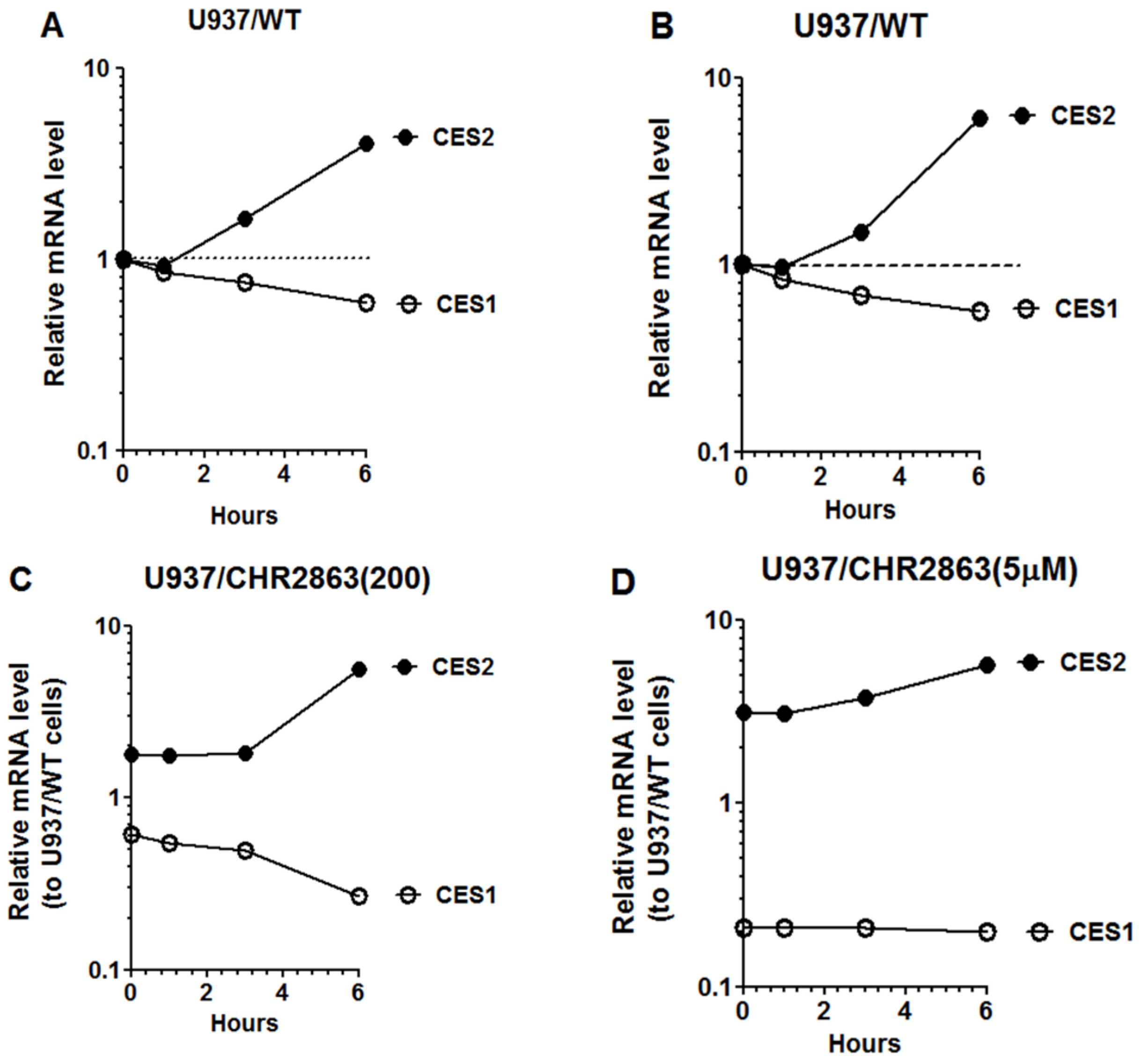

Figure S3: Dynamics of CES1 and CES2 mRNA expression following 0-6 hours exposure of **(A)** U937/WT cells + 50 nM CHR2863, **(B)** U937/WT + 6 µM CHR2863, **(C)** U937/CHR2863(200) + 6 µM CHR2863, and **(D)** U937/CHR2863(5µM) cells + 6 µM CHR2863. Results are expressed relative to values of U937/WT at t=0, and depict the mean of two experiments performed in triplicate.

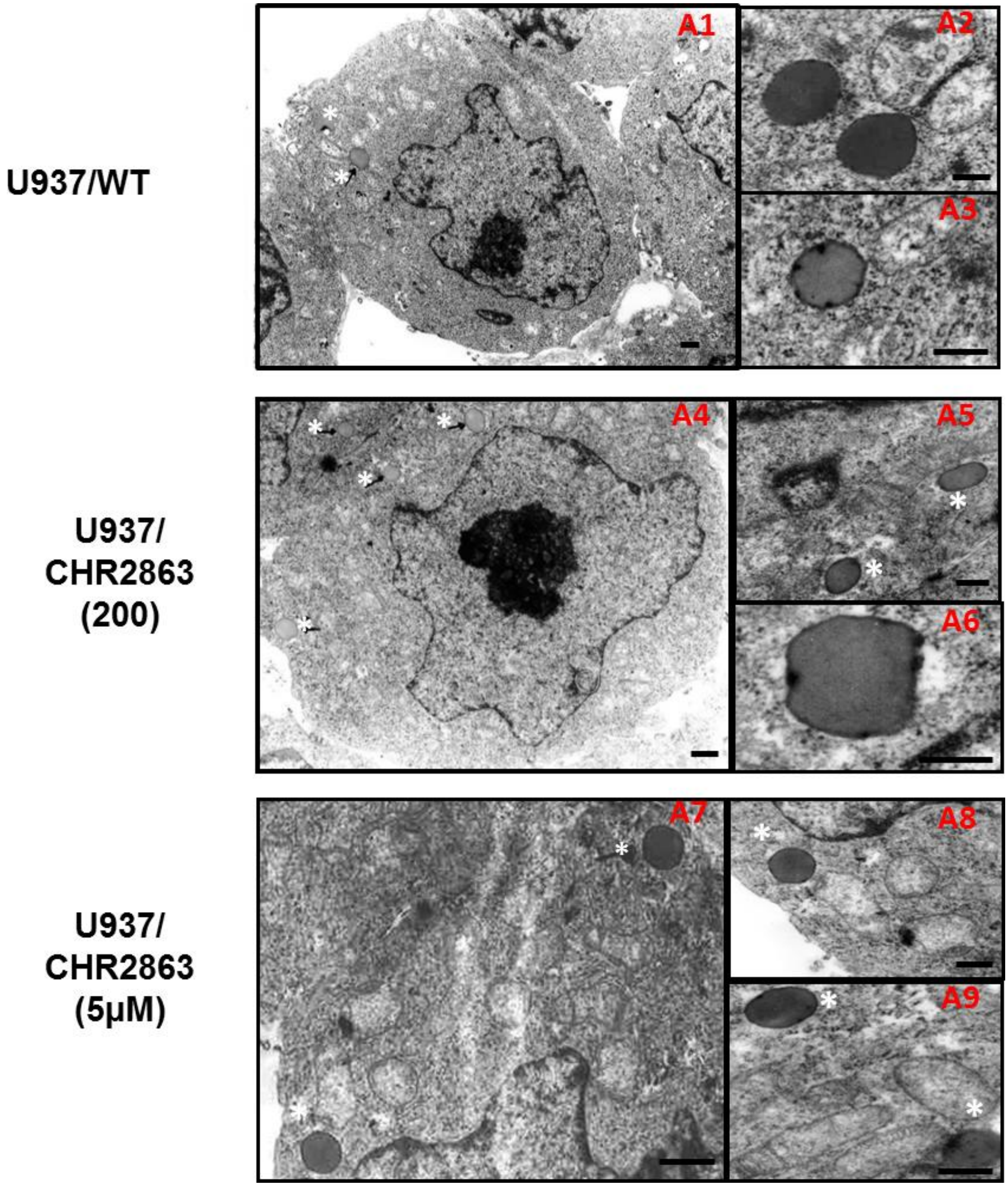

Figure S4. Transmission electron microscopy identification of lipid droplets (indicated by *) in U937/WT, U937/CHR2863(200) and U937/CHR2863(5uM) cells. Magnification; 4,000-60,000 fold. Bar = 1000 nm (Figure A1, A4, A7), bar = 500 nm (Figure A2, A3, A5, A6, A8, A9).

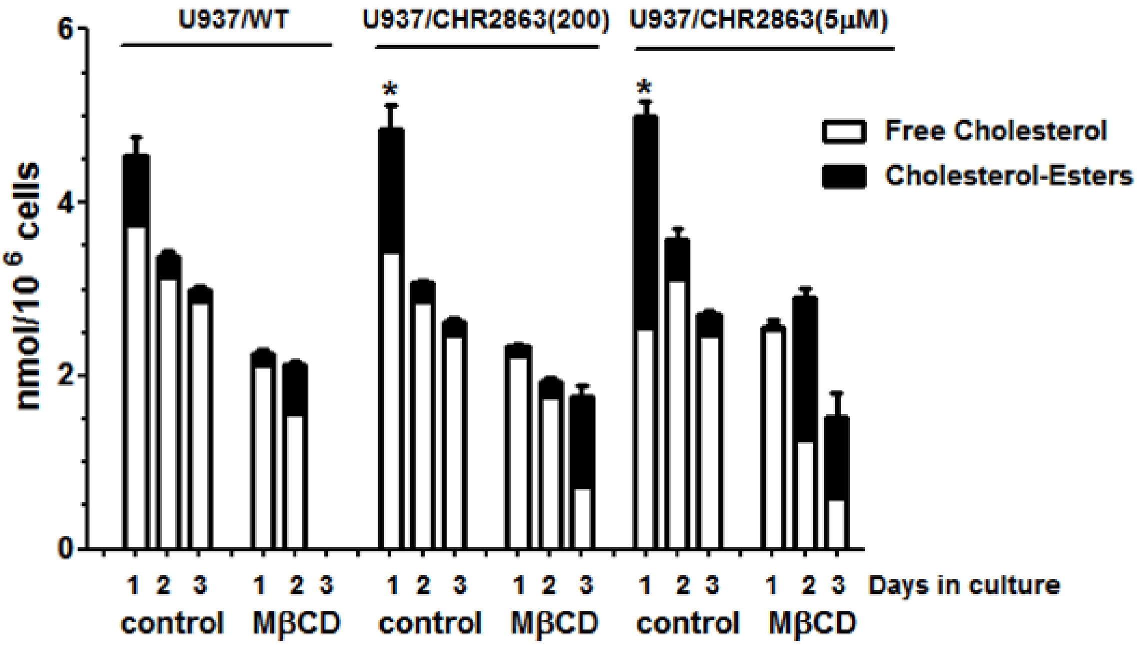

Figure S5: Free cholesterol and cholesteryl ester content in U937/WT, U937/CHR2863(200), and U937/CHR2863(5µM) cells during 3 days cell culture, with, for the CHR2863-resistant cells, in the presence of their selective concentrations of CHR2863. As a control experiments were performed in the presence of 1 mM of cholesterol-lowering drug methyl-β-cyclodextrin (MβCD). Results are the mean ± SD of 3 separate experiments performed in duplicate.

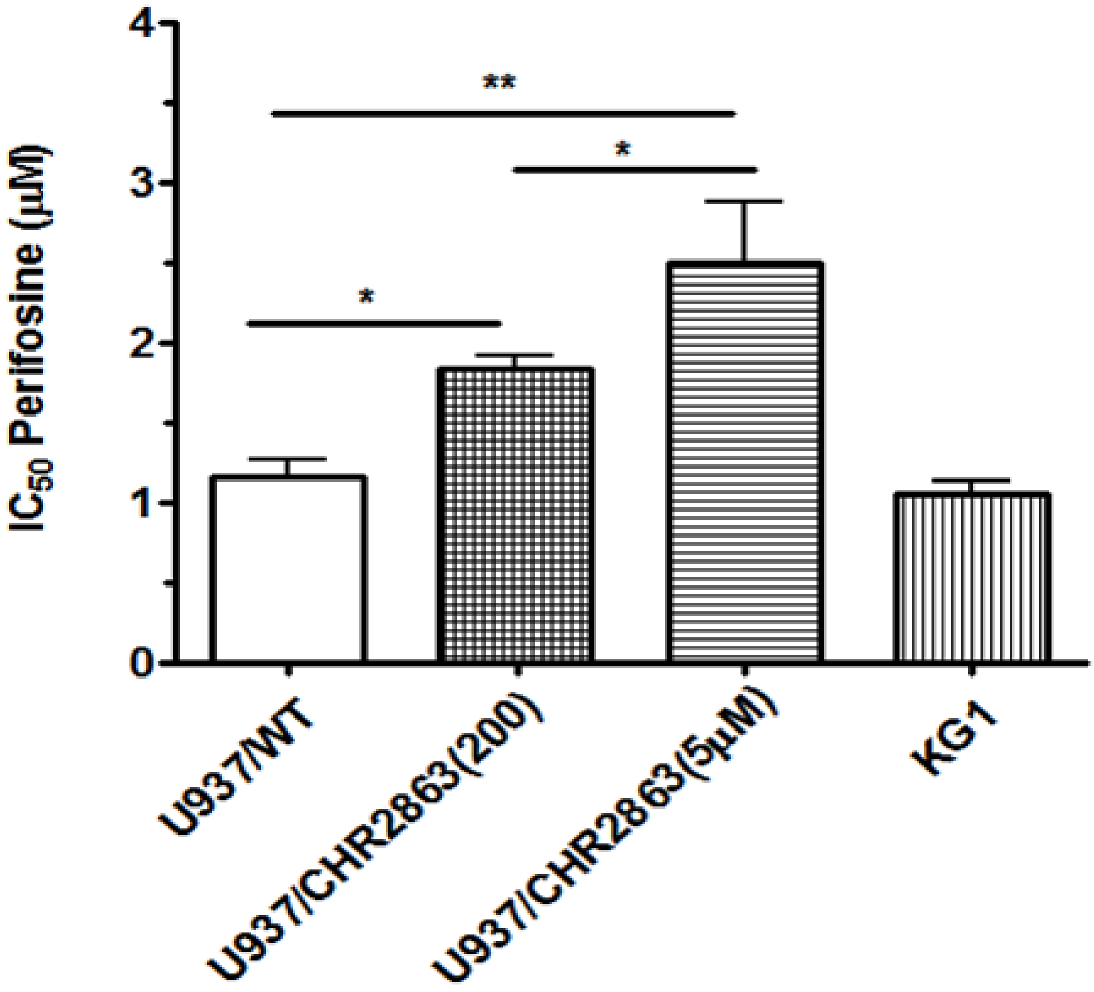

Figure S6: Growth inhibitory effects of the Akt inhibitor Perifosine against U937/WT, U937/CHR2863(200), U937/CHR2863(5µM) cells and KG1 cells (as reference). Results are expressed as mean (± SD) of 3-4 separate experiments. (*): $p < 0.05$.
